# Unraveling the frequency of intestinal constipation in woman with urinary incontinence: a descriptive observational study

**DOI:** 10.61622/rbgo/2025rbgo42

**Published:** 2025-07-15

**Authors:** Bruna Isadora Thomé, Karoleen Oswald Scharan, Gisela Maria Assis, Auristela Duarte de Lima Moser

**Affiliations:** 1 Pontifícia Universidade Católica do Paraná Curitiba PR Brazil Pontifícia Universidade Católica do Paraná, Curitiba, PR, Brazil.; 2 Universidade Federal do Paraná Curitiba PR Brazil Universidade Federal do Paraná, Curitiba, PR, Brazil.

**Keywords:** Urinary incontinence, Urinary incontinence, stress, Constipation, Women's health, Comprehensive health care, Quality of life, Life style, Surveys and questionnaires

## Abstract

**Objective:**

To identify the frequency of functional constipation in women with urinary incontinence.

**Methods:**

A cross-sectional, quantitative, descriptive, and exploratory study was conducted from September 2019 to January 2020 with 227 women (over 18 years old) at the *Hospital de Clínicas, Universidade Federal do Paraná*. A structured form collected sociodemographic and general health data, while bowel habits were assessed using the Rome IV criteria, the Bristol Stool Scale, and the International Consultation on Incontinence Questionnaire – Short Form (ICIQ-SF). Comparisons between women with and without constipation involved χ^2^, Fisher's Exact, Student's t-test, Mann-Whitney U, and univariate logistic regression was performed to assess the association between sociodemographic/personal factors and the risk factor constipation. All women had a clinical diagnosis of urinary incontinence, and the analysis also compared those with and without constipation. Odds ratios and their respective 95% confidence intervals were estimated for each univariate model. A 5% significance level was adopted.

**Results:**

The participants had a median age of 62 years (range 23–97). Functional constipation was identified in 80.2%(n=182), and mixed urinary incontinence was predominant in this group (88.5%, n=161). Physical activity emerged as a protective factor against constipation (OR=0.47; 95% CI=0.22–1.01; p=0.05), though only 22.5%(n=41) reported regular exercise. Conclusion: The high frequency of functional constipation in women with urinary incontinence highlights a significant impact on quality of life and underscores the importance of integrated, conservative therapeutic strategies, including early lifestyle interventions such as regular physical activity, to prevent worsening of both conditions. Longitudinal investigations are recommended.

## Introduction

Urinary incontinence (UI) and functional constipation (FC) are characterized by a set of symptoms that represent changes in the functions of the genitourinary and digestive systems, respectively.

Urinary incontinence is the involuntary loss of urine and is currently divided into three subtypes. There is stress urinary incontinence (SUI), where urine is lost during exertion, sneezing, or coughing; urgency urinary incontinence (UUI), in which the loss is immediately preceded or accompanied by the urgency to urinate; and mixed urinary incontinence (MUI), which presents symptoms of the previous subtypes.^(1,2)^

Functional constipation may present with the decreased bowel movement frequency, abdominal pain, palpable stool in the abdomen and rectal vault, and the stool being large, hard, or in small pieces.^(3–5)^

Both conditions affect females more frequently across various age groups. Urinary incontinence affects approximately 25 to 45% of women worldwide and is considered a public health issue due to its physical, psychological, and social impacts, as well as the individual and public costs to healthcare systems.^(6,7)^ Although not a natural part of aging, the prevalence of UI increases with age for both sexes.^(6)^

Constipation affects about 20% of the global adult population, with a frequency of 34% in older adults, including the Brazilian population.^(3,4)^

Abnormal stimuli from pelvic organs, such as rectal distension caused by constipation, can compress the bladder due to anatomical proximity and shared innervation, resulting in urinary urgency, emptying difficulties, and alterations in detrusor muscle contractions.^(8,9)^

The functional connection between the bladder and bowel could have implications for the management of pelvic disorders, as treating an altered condition in the bladder may affect bowel function and vice versa. Thus, treatment for chronic constipation has a high likelihood of reducing urinary symptoms, while treatment for urinary symptoms with antimuscarinic medication may promote constipation.^(10)^ Furthermore, combined treatments for both conditions, along with increased fluid intake, may improve both health conditions.^(4,5,10)^

The relationship between UI and vaginal delivery, multiparity, short intervals between pregnancies, newborn weight, forceps use, etc., is well-established in the literature.^(6,8)^ Evidence suggests that constipation could influence the development of UI.^(7,11)^

The alterations in bodily functions mentioned above negatively impact women's quality of life (QoL), directly interfering with daily activities, restricting participation in physical activities, work, household chores, personal hygiene, and sexual relationships, for example.^(6,7)^

Understanding and evaluating these impacts can contribute to the development of better strategies for diagnosing, treating, and preventing both UI and FC, whether as isolated or combined health conditions.^(10,12)^

The present study aimed to identify the frequency of functional constipation in women with urinary incontinence.

## Methods

This quantitative, observational, cross-sectional study collected data from the voiding dysfunction outpatient clinics, incontinence/nursing clinics, and the interdisciplinary pelvic floor group at the *Hospital de Clínicas of the Universidade Federal do Paraná* (UFPR), between September 2019 and January 2020. Women over 18 years old with UI symptoms were included, while women with anatomical alterations and/or neurological dysfunctions that could lead to urinary incontinence and/or functional constipation were excluded. The sample size calculation was based on the results of a pilot study, considering a significance level of 5% and a statistical power of 90% resulting in the participation of at least 180 subjects. For the pilot study, a sample of 24 participants was calculated using Epitools software (https://epitools.ausvet.com.au/), based on the proportion of FC cases among individuals with UI, which is reported in the literature to be around 55%, with a margin of error of 23% and a confidence level of 95%. The studies showed a prevalence of 79% of FC, considering a significance level of 5% and a statistical power of 90%. Recruitment occurred through simple random sampling of participants from the daily appointment list organized by the outpatient clinic. Through an individual 30-minute interview, a single evaluator administered the following tools: a sociodemographic and general health structured form, an intestinal habit questionnaire (IHQ), and the Portuguese-translated and validated version of the International Consultation on Incontinence Questionnaire - Short Form (ICIQ-SF).

### Rome IV criteria

A questionnaire to assess intestinal function and the presence of functional constipation in participants, supported by the Bristol Stool Scale for stool consistency to descriptively evaluate the shape of the fecal content. The onset of symptoms should occur at least 6 months prior to diagnosis, and they should be present for the last 3 months. The presence of FC will be confirmed with the indication of two or more statements from this list.^(13)^

### International Consultation on Incontinence Questionnaire - Short Form (ICIQ-SF)

A questionnaire designed to assess the impact of urinary incontinence on quality of life and the severity of reported urinary loss. It consists of four questions that evaluate the frequency, severity, and impact of UI, with scores in increasing order of severity, generating a final score with a maximum of 21 points. Additionally, it includes a set of eight self-diagnosis items related to the causes or situations of UI experienced by the subjects. The classification of UI subtypes (SUI, MUI, UUI) among participants was determined by analyzing seven of the eight statements from item 6 of the ICIQ-SF questionnaire. Participants who answered "yes" to at least one of statements 3, 5, 7, or 8 were classified as having SUI. Those who answered "yes" to at least one of statements 2, 4, or 6 were classified as having UUI. If participants endorsed one or more statements from both subtypes, they were classified as MUI. Statement 1 was not used because it indicates no urinary leakage at any time.

### Statistical analysis

The IBM SPSS statistical program, version 25, was used. Results for categorical variables were described in absolute and relative frequency, and results for quantitative variables were described by mean and standard deviation, median, and range. To estimate parameters of interest, 95% confidence intervals were constructed. The comparison of the frequency of FC between the three UI subgroups was performed using the Pearson chi-square test. The identification of factors that predispose to the association between FC and UI was analyzed using logistic regression, considering FC as the dependent variable and sociodemographic and personal factors as independent variables. All women had a clinical diagnosis of UI, and the analysis compared those with and without constipation. For each univariate model, odds ratios (OR) and their respective 95% confidence intervals were estimated. A 5% significance level was adopted.

## Results

A total of 227 women participated in the study, with a mean age of 60.33±12.26 years. The frequency of FC in the sample was 80.2%(n=182), as shown in table 1.

**Table 1:** Sociodemographic characteristics and gestational profile of the sample with or without functional constipation

Variables	Population n=227 n(%) or Median[Min – Max]	With constipation n=182 n(%) or Median [Min – Max]	Without constipation n=45 n(%) or Median [Min – Max]	p-value
Age	62[23-97]	60.5[23-97]	63[38-80]	0.462*
Adult young	2(9)	2(1)	0	
Adult	96(42.3)	82(41.4)	14(29.8)	
Older adults	129(56.8)	98(49.5)	31(66)	
Education Level				0.684**
Illiterate	10(4.4)	9(4.9)	1(2.2)	
Primary school	135(59.5)	105(57.7)	30(66.7)	
High school	65(28.6)	54(29.7)	11(24.4)	
Higher Education	17(7.5)	14(7.7)	3(6.7)	
Employment Status				0.655**
Homemaker	138(60.8)	108(59.3)	30(66.7)	
Retired	5(2.2)	4(2.2)	1(2.2)	
Paid Employment	84(37)	70(38.5)	14(31.1)	
Gestational Profile				
Number of Pregnancies	3[2 – 5]	3[2 – 5]	3[2.5 - 5]	0.841****
Vaginal Deliveries	2[1 – 3]	2[1 – 3]	2[2 – 3.5]	0.605****
Cesarean Sections	0[0 – 1]	0[0 – 1]	1[0 – 1]	0.451****
Abortions	0[0 – 1]	0[0 – 1]	0[0 – 1]	0.182****
Episiotomy	146(64.3)	112(61.5)	34(75.6)	0.085***
Forceps	39(17.2)	29(15.9)	10(22.2)	0.377***

SD - standard deviation;

* Student's t-test for independent measures,

** Pearson's chi-square test,

*** Fisher's Exact Test,

**** Mann-Whitney U test; significance level p ≤ 0.05

The education level was predominantly low, with 59.5%(n=135) of the sample having primary school and 4.4%(n=10) of women being illiterate. Regarding occupational activity, the highest percentage was homemakers (60.8%, n=138). The distribution of education level and occupational activity was similar between the groups with and without FC. The number of pregnancies had a median of 3 for both groups, with a median of 2 vaginal deliveries. The variables episiotomy and forceps use had incidences of 64.3%(n=146) and 17.2%(n=39), respectively. These variables did not show significance in the comparison test with FC. The most prevalent positive Rome IV criteria among participants with FC were straining during defecation (89.6%, n=163), sensation of incomplete/unsatisfactory evacuation (84.1%, n=153), and hard or irregular stools (76.4%, n=139). These were followed by sensation of pain or obstruction (74.2%, n=135), manual maneuvers (65.4%, n=119), rarely having loose/watery stools without laxative use (63.7%, n=116), and fewer than three bowel movements per week (24.2%, n=44). Table 2 presents the health conditions, intestinal profile, and lifestyle habits of the studied sample as well as the univariate logistic regression analysis.

**Table 2:** Health condition, lifestyle habits, and intestinal profile of the sample by groups with or without IC; univariate logistic regression for sociodemographic and personal factors

Variables	Population n=227 %(±SD)	With constipation n=182 %(±SD)	Without constipation n=45 %(±SD)	p-value	Univariate logistic regression OR (CI95%) p-value
Menopause	183(80.6)	148(81.3)	35(77.8)	0.674*	1.23 (0.49-3.11) 0.66
History of pelvic surgeries	183(80.3)	143(78.6)	40(88.9)	0.142*	0.42 (0.14-1.24) 0.18
Smoker	28(12.3)	25(13.7)	3(6.7)	0.310*	2.21 (0.59-8.25) 0.24
Former smoker	56(24.7)	45(24.7)	11(24.4)	1.000*	1.15 (0.50-2.63) 0.74
NCDs	160(70.5)	129(70.9)	31(68.9)	0.856*	0.53 (0.17-1.62) 0.26
Respiratory problems	44(19.4)	39(21.4)	5(11.1)	0.142*	2.37 (0.75-7.54) 0.14
Systemic arterial hypertension	121(53.3)	99(54.4)	22(48.9)	0.511*	2.93 (0.06-154.87) 0.60
Diabetes	49(21.6)	42(23.1)	7(15.6)	0.317*	3.55 (0.12-101.94) 0.46
Others	22(9.7)	18(9.9)	4(8.9)	1.000*	0.93 (0.24-3.63) 0.92
Continuous-use medication	188(82.8)	149(81.9)	39(86.7)	0.515*	0.54 (0.16-1.83) 0.33
Diuretic	67(29.5)	51(28)	16(35.5)	0.362*	0.47 (0.18-1.22) 0.12
Hormone replacement therapy	9(4)	5(2.7)	4(8.9)	0.079*	0.56 (0.13-2.53) 0.45
Antihypertensive	120(52.9)	98(53.8)	22(48.9)	0.618*	1.05 (0.02-53.99) 0.98
Antidepressant	83(36.6)	70(38.5)	13(28.9)	0.300*	1.63 (0.71-3.73) 0.25
Oral hypoglycemic agents	47(20.7)	40(22)	7(15.6)	0.415*	0.41 (0.01-11.76) 0.60
Others	72(31.7)	61(33.5)	11(24.4)	0.687**	1.08 (0.69-1.68) 0.75
Engage in physical activity	58(25.6)	41(22.5)	17(37.8)	0.055*	0.47 (0.22-1.01) 0.05
Intestinal profile					
Evacuationdays/week	5.44 (±0.14)	5.12(±0.16)	6.73 (±0.88)	-	-
Time to evacuation in minutes Median (Min-Max]	5[3 – 10]	5.5[5 – 15]	3[3 – 5]	-	-
Use of laxatives (%)	121(53.3)	116(63.7)	5(11.1)	-	-

SD - standard deviation; NCDs - Noncommunicable Diseases;

* Fisher's Exact Test

** Pearson's Chi-square Test. significance level p≤0.05

Table 2 highlights the high prevalence of systemic arterial hypertension (53.3%) and the use of continuous medications (82.8%). Smoking had a low prevalence among the evaluated women (12.3%), with a slightly higher percentage among those with constipation (13.7%). Regarding physical activity, only 25.6% of the study population met the minimum recommendation of 150 minutes per week. In the group with FC, this percentage was lower (22.5%) compared to women without FC who engaged in physical activity (37.8%). This was the only variable that showed statistical significance (p=0.055) in the comparison between groups. After univariate logistic regression, this variable was identified as a protective factor for the development of FC (OR= 0.47; CI= 0.23–0.96, p=0.05), supporting the observed difference between groups with and without FC. Women with FC had an average of 5.12 ±0.16 days per week, with a bowel movement duration of 5.5 [5-15] minutes.

Table 3 shows the frequency of the three subtypes of UI in groups with and without FC, the use of protective pads in underwear and ICIQ-SF results for each question and the total score. Mixed incontinence was the most frequent subtype in both groups. The second most common type of UI was urgency UI (7.1%) among women with FC, whereas in the group without FC, the prevalence was the same for SUI and UUI (8.9% each). The association between the three UI subtypes and the presence of FC did not reach statistical significance. Women with both UI and FC were the ones who most frequently used protective pads to manage urine leakage (72.5%). Regarding the ICIQ-SF score, which assesses the impact of UI on quality of life, the mean score for the study population was 14.29 ± 0.30. Among women with constipation, the score was 14.68 ± 4.27, while for those without constipation, it was 12.73 ± 5.25. A statistically significant difference was observed between the groups (p = 0.04).

**Table 3:** ICIQ-SF: Characteristics of urine leakage, final score, and distribution of incontinence subtypes by groups with or without IC

Type of UI	With constipation n=182 (%)	Without constipation n=45 (%)	p-value
Mixed urinary incontinence	161(88.5)	37(82.2)	0.430*
Urgency urinary incontinence	13(7.1)	4(8.9)	0.430*
Stress urinary incontinence	8(4.4)	4(8.9)	0.430*
Protective linings for UI	132(72.5)	29(64.4)	-
ICIQ-SF Median[Min-Max] or Media±SD			
Q1	4[1-5]	2[1-5]	-
Q2	2[1-3]	1[1-3]	-
Q3	8[0-10]	8[0-10]	-
Final score	14.7±4.3	12.7±5.3	0.04**

UI- urinary incontinence;

* Pearson's chi-square;

** Fisher's Exact Test; Significance level p≤0.05

## Discussion

This study presented data from women already diagnosed with UI, revealing a high frequency of intestinal constipation among them (80.2%). It can be observed that, despite variations in age range and constipation prevalence across studies, FC is a factor that should be considered in the context of UI. This perspective is supported by the findings of a meta-analysis that examined 16 studies on the relationship between constipation and the risk of urinary incontinence. The authors concluded that FC is significantly associated with UI in women, and therefore, approaches aimed at improving FC should be considered for this population.^(14)^

Aging is not exclusively related to the development of FC; however, changes in lifestyle habits over time may increase the predisposition to this dysfunction.

The level of education predominantly showed a lower range (59.5% with primary school and 4.4% illiterate), a finding similar to that observed in other studies.^(11,12)^ In the present study, it was observed that women often lacked knowledge about their own health conditions and had difficulty following the guidance provided to them. Therefore, it is essential that professionals working with this population consider the level of education and adopt different strategies for the conservative management of UI and FC.

The findings of this study regarding the number of pregnancies (median of 3) and types of delivery (vaginal) are consistent with studies conducted in the Brazilian states of Piauí^(11)^ and Minas Gerais.^(15)^ In this context, the variables episiotomy and forceps use in this study showed a prevalence of 64.3% and 17.2%, respectively. It is important to consider these variables in conjunction with the presence of constipation in this population of women with UI to better understand the factors that contributed to these symptoms and determine the most appropriate treatment approach.

Regarding the Rome criteria present in the FC group of this study, it was observed that the most frequently mentioned criteria by the participants were straining during defecation (89.6%), a sensation of incomplete/unsatisfactory evacuation (84.1%), and hard or irregular stools (76.4%). These data align with a study conducted in Turkey^(16)^ which found that the most frequent criteria were straining during defecation (72%), hard or irregular stools (65%), and a sensation of incomplete/unsatisfactory evacuation (56%) in the group experiencing associated urinary loss. These characteristics are often associated with inadequate water intake, low fiber consumption, and high intake of processed foods.^(5)^

Changes in lifestyle habits could contribute to conservative measures to improve intestinal constipation, as neglecting the urge to defecate, often due to the overload of daily tasks, contributes to conditions that impair intestinal motility and favor constipation.

According to the lifestyle habits and health conditions observed in this study, a high frequency of systemic arterial hypertension (SAH) (53.3%) and frequent use of continuous medications (82.8%) were identified. Although these factors are common, no significant correlation with FC was established.

The predominant pattern of medications included antihypertensives, diuretics, and antidepressants, with the latter being more common among individuals with FC. The analysis of medication use highlighted the vulnerability associated with polypharmacy and emphasized the importance of understanding the patient's medical history, comorbidities, and drug interactions.

Self-medication or prescription without a comprehensive understanding of overall health can contribute to the worsening or chronicity of FC. It is worth noting that although commonly used to treat urinary symptoms, antimuscarinics can induce constipation, emphasizing the need for a cautious approach in patients with these symptoms.^(16)^

These data highlight the importance of a global observation, considering the proximity and interference between the urinary bladder and the intestine when evaluating lower urinary tract symptoms, to outline more comprehensive and targeted treatment strategies, tailored to the woman's cognitive capacity and daily life limitations, as managing constipation can positively impact urinary symptoms.

The practice of physical activity was not prevalent in this study, with a rate of 25.6%, lower than the 37.8% observed in women without FC who engaged in physical activities (37.8%). This variable emerged as a protective factor against the development of FC, a finding consistent with previous research indicating that physical activity, good nutrition, and adequate fluid intake contribute to the improvement of motility and intestinal transit time.^(3)^

The approach to assessing weekly bowel movement frequency proved particularly intriguing in this study. When participants were asked about their bowel habits, many initially denied having problems, claiming to defecate almost daily. However, upon applying the Rome criteria, most participants exhibited signs and confirmation of FC, even when averaging 5.12 ± 0.16 bowel movements per week. These signs included straining during defecation, a sensation of incomplete/unsatisfactory evacuation, hard or irregular stools, a feeling of pain or obstruction, manual maneuvers, loose/watery stools rarely occurring without the use of laxatives, and fewer than three bowel movements per week.

These data show that most participants associated FC with the act of not defecating. This finding also reinforces the importance of raising awareness and providing education on the topic to patients, which may be further influenced by the low educational level observed in our sample.

In this study, mixed urinary incontinence (MUI) was the most prevalent type in both the group with FC (88.5%) and the group without FC (82.2%), compared to stress and urgency urinary incontinence, with a mean age of 60 years. Research indicates that age plays a role in the distribution of UI types, with MUI and urgency urinary incontinence (UUI) predominating among older and middle-aged women, while stress urinary incontinence (SUI) is more common in young adults.^(6,9)^ These findings are consistent with a previous study involving young adult women, where SUI was more prevalent.^(17)^

A study conducted in Brazil reported a similar distribution, with MUI (58%), UUI (16.8%), and SUI (25.2%) among the women evaluated,^(12)^ reinforcing that understanding the distribution of UI subtypes, along with identifying the presence or absence of associated FC, can more effectively guide treatment options for managing UI, prioritizing the symptoms that are most bothersome for women.

Research has sought to gain a more detailed understanding of the impact of these dysfunctions on QoL and to assess their significant interference with individuals’ productive activities and social interactions.^(7,18)^

In the present study, we were able to reflect on this through the ICIQ-SF Score, which demonstrated a significant decline in QoL when associated with FC (14.68 ± 4.27). This low QoL score, especially when UI is associated with FC, highlights the risk of underestimating the importance of intestinal dysfunction.

The ICIQ-SF itself, in one of its questions, uses a Likert scale to assess how much urine leakage interferes with daily life. Combined with a lack of knowledge about this dysfunction and feelings of embarrassment, many women refrain from seeking healthcare services and professional help.^(19)^

These women tend to be more likely to seek assistance when symptoms significantly interfere with their social, physical, religious activities, and sexual relationships. These determining factors underscore the importance of considering the real impact of UI on women's lives to encourage the pursuit of professional care.

The QoL findings in the present study highlight the importance of viewing signs and symptoms not merely as issues to be treated and resolved but as indicators that warrant exploration of their broader impact on individuals’ lives. This approach aligns with an expanded concept of health, in which physical, structural, social, and environmental domains are considered to understand the lived health condition and to develop care protocols that address all aspects of health.

## Conclusion

The high frequency of FC in women with UI highlights a significant impact on quality of life and underscores the importance of integrated, conservative therapeutic strategies, including early lifestyle interventions such as regular physical activity, to prevent worsening of both conditions. Longitudinal investigations are recommended.

## References

[1] D’Ancona C, Haylen B, Oelke M, Abranches-Monteiro L, Arnold E, Goldman H (2019). The International Continence Society (ICS) report on the terminology for adult male lower urinary tract and pelvic floor symptoms and dysfunction. Neurourol Urodyn.

[2] Squillace MC, Alorda MB, Cafferatta S, Carlucci ML, Escobar MM, Patiño SS (2023). [Nutritional risk factors associated with urinary incontinence and its impact on quality of life in adult women]. Actual Nutr.

[3] Dantas AA, Barbosa IR, Castro SS, Ferreira CW, Camara SM, Dantas DS (2020). Prevalence and factors associated with constipation in premenopausal women: a community-based study. Arq Gastroenterol.

[4] Silva AK, Pereira PM, Seixas TB, Percegoni N (2020). [Intestinal constipation and factors associated in patients inside in a university hospital]. Rev Assoc Bras Nutr - RASBRAN.

[5] Rollet M, Bohn T, Vahid F (2021). Association between dietary factors and constipation in adults living in Luxembourg and taking part in the ORISCAV-LUX 2 Survey. Nutrients.

[6] Alves CA, Ferreira DC, Lima MF, Coimbra KA, Vaz CT (2022). Prevalence of urinary incontinence. impact on quality of life and associated factors in users of Primary Health Care Units. Fisioter Mov.

[7] Alencar-Cruz JM, Lira-Lisboa L (2019). [Impact of urinary incontinence on quality of life and its relationship with symptoms of depression and anxiety in women]. Rev Salud Pública (Bogota).

[8] Ribeiro GL, Firmiano ML, Vasconcelos CT, Vasconcelos JA, Lopes MH, Damasceno AK (2023). Knowledge, attitude, and practice of pregnant women about urinary incontinence: observational study. Estima.

[9] Pereira LC, Silva JP, Lima CR, Ferreira CW (2022). Prevalence, knowledge, and factors associated with urinary incontinence in female students of a physical therapy undergraduate course. Fisioter Pesqui.

[10] Santos AC, Dias SN, Delgado A, Lemos A (2024). Effectiveness of group aerobic and/or resistance exercise programs associated with pelvic floor muscle training during prenatal care for the prevention and treatment of urinary incontinence: a systematic review. Neurourol Urodyn.

[11] Braga FC, Benício CD, Bezerra SM, Silva A, Costa AQ, Santos ES (2021). Profile of patients with urinary incontinence in a university outpatient clinic. Estima.

[12] Méndez LM, Moura AC, Cunha RM, Figueiredo VB, Moreira MA, Nascimento SL (2022). Behavioral therapy in the treatment of urinary incontinence: quality of life and severity. Fisioter Mov.

[13] Taniguchi TM, Abreu GE, Portugal MM, Barroso U (2022). Cross-cultural adaptation and validation of the constipation scoring system for the Brazilian population. Arq Gastroenterol.

[14] Lian WQ, Li FJ, Huang HX, Zheng YQ, Chen LH (2019). Constipation and risk of urinary incontinence in women: a meta-analysis. Int Urogynecol J.

[15] Gonçalves JL, Castro BN, Castro EM, Diniz MB (2022). [Prevalence of urinary incontinence in the ambulatory service of Gynecology and Obstetrics]. Rev Interdiscip Ciênc Méd.

[16] Akan S, Yilmaz O, Ediz C, Yenigurbuz S, Yesildal C (2021). Effects of the constipation treatment in women who have overactive bladder and functional constipation. Ann Med Res.

[17] Carvalho C, Serrão PR, Beleza AC, Driusso P (2020). Pelvic floor dysfunctions in female cheerleaders: a cross-sectional study. Int Urogynecol J.

[18] Pizzol D, Demurtas J, Celotto S, Maggi S, Smith L, Angiolelli G (2021). Urinary incontinence and quality of life: a systematic review and meta-analysis. Aging Clin Exp Res.

[19] Cavenaghi S, Lombardi BS, Bataus SC, Machado BP (2020). [Effects of physiotherapy on female urinary incontinence]. Rev Pesqui Fisioter.

